# Impact of school salad bars on fruit and vegetable selection, intake, and waste in Mid-Atlantic elementary schools

**DOI:** 10.1186/s12966-025-01713-y

**Published:** 2025-02-05

**Authors:** Melanie K. Bean, Suzanne E. Mazzeo, Lilian de Jonge, Laura Thornton, Hollie Raynor, Ashley Mendoza, Sarah Farthing, Bonnie Moore

**Affiliations:** 1 Department of Pediatrics, School of Medicine, Children’s Hospital of Richmond at Virginia Commonwealth University, Box 980140, Richmond, VA 23298 USA; 2 Department of Psychology, Virginia Commonwealth University, Box 842018, Richmond, VA 23284 USA; 3 Department of Nutrition and Food Studies, George Mason University College of Public Health, 4400 University Dr. MSN 1F7, Fairfax, VA 22030 USA; 4 Department of Psychiatry, University of North Carolina at Chapel Hill, Chapel Hill, NC 27599 USA; 5 Department of Nutrition, University of Tennessee, 1215 W. Cumberland Ave., Knoxville, TN 37996 USA; 6 Real Food for Kids, 6166 Hardy Drive, McLean, VA 22101 USA

**Keywords:** Salad bar, Plate waste, Fruit and vegetable, Elementary school, National School Lunch Program

## Abstract

**Background:**

Few studies have empirically examined the impact of school salad bars on elementary students’ fruit and vegetable (FV) consumption within the National School Lunch Program (NSLP). This natural experiment evaluated the impact of salad bars on FV selection, intake, and waste within elementary schools.

**Methods:**

Seven school pairs, matched on Title I status and percentage of students from ethnic or racial minority backgrounds, were randomly selected. All schools served pre-portioned FV at baseline. One school within each pair received a salad bar; the other continued to serve pre-portioned FV (Control). Digital imagery plate waste methods were applied at baseline and 4–6 weeks after schools installed salad bars (post). Images were rated in the laboratory (ICCs = .94-.99) to determine FV selection, intake, and waste (servings [1 NSLP serving = ½c]). Multilevel modeling evaluated group (Salad Bar vs Control) and time (baseline vs post) differences and group-by-time interactions. Differences in outcomes by Title I status were also examined.

**Results:**

Across schools, mean NSLP participation was 54%. *N* = 6,623 trays were included (*n* = 3,273 Salad Bar; *n* = 3,350 Control). Students in Salad Bar schools selected (+ .44c) and consumed (+ .36c) more FV at post, compared to baseline. Control students decreased FV selection (-.05c) with no change in intake from baseline to post. Group, time, and group-by-time interactions were significant (ps < .0001). When examined separately, results suggest that these effects are driven by fruit. Salad Bar students increased fruit selection (+ .45c), intake (+ .36c), and waste (+ .09c) from baseline to post; no significant changes were observed in Controls. There was no significant change in vegetable selection, intake or waste for either group. Findings did not differ based on Title I status.

**Conclusions:**

Salad bars were effective in increasing elementary school students’ fruit selection and intake, yet did not increase vegetable selection or intake. Additional efforts are needed to increase vegetable intake and minimize fruit waste from salad bars. Consistent findings across schools, regardless of Title I status, suggest potential for salad bars to yield increased fruit intake across socioeconomic groups. Findings can inform policies designed to increase FV intake within the NSLP.

**Trial registration:**

This investigation reports results of a systematic evaluation of school salad bars and does not meet criteria for a clinical trial, yet was retrospectively registered (10/28/22) in clinicaltrials.gov (NCT05605483) as an observational study.

**Supplementary Information:**

The online version contains supplementary material available at 10.1186/s12966-025-01713-y.

## Background

There is an urgent need to enhance understanding of factors that can improve children’s fruit and vegetable (FV) intake, given the well-established links between FV consumption and chronic illness, including cardiovascular disease and cancer [1, 2]. Moreover, children living in neighborhoods characterized by high poverty consume fewer FVs than their peers and are also the most likely to participate in the National School Lunch Program (NSLP), a federally-assisted meal program serving 30 million students daily [3–5]. The NSLP mandates meal pattern requirements, including that students must select a fruit and/or vegetable at lunch each day. Thus, the school food environment is a critical target of nutrition-related public health efforts.

School salad bars are an intuitively appealing approach to increasing students’ FV intake [6, 7]. Consequently, significant financial resources have been invested in the installation of salad bars in schools. Yet it is not known if these efforts have yielded the expected effects on children’s FV consumption [6]. Although few studies have empirically investigated this issue, the limited available data raise some concerns. For example, although a large majority (85%) of elementary students in one study reported enjoying the opportunity to select FVs from their school salad bars, a much smaller proportion (44%) actually used the salad bar at least once per week [8]. Results of a another investigation conducted with elementary students indicated that FV consumption was not significantly different in schools with salad bars compared with schools serving only pre-portioned FVs [9]. However, this research was conducted ~ 20 years ago, and might not be generalizable in the current school cafeteria climate, especially given the many NSLP changes implemented since that time. For example, current NSLP guidelines mandate that schools serve a variety of vegetables each week and require students take at least one serving of fruit and/or vegetables daily; however, these mandates were not in place prior to 2012. Moreover, much of the extant research investigating the impact of school salad bars on students’ FV intake is limited by a lack of longitudinal and objective data. For example, middle and high school students have reported consuming more FVs in schools with salad bars [10–12]. However, in these investigations, FV intake was exclusively measured via self-report, a method more vulnerable to bias than objectively measured consumption. Slusser and colleagues [13] reported that FV intake increased after salad bar installation (+ 1.1 servings per day). However, these investigators also relied on self-report data and did not evaluate fruit and vegetable consumption separately. In addition, the two-year time lag between baseline and post assessments and the 30% student transience rate likely decreased the probability that the same children were assessed at both time points, and introduced other potential history effects that could impact outcomes (e.g., school climate changes).

A more recent study evaluated FV intake before and one month after installation of salad bars in two Title I elementary schools (> 95% Black, 100% of students received free meals) [14]—Title I designation indicates that at least 40% of students within a school are from low-income families, making the entire school eligible for federal funding. Objective, digital imagery plate waste methods indicated that students selected more different *types* of FVs after the introduction of salad bars. However, at post, self-served FV portions were significantly smaller than those served by food service personnel, and mean FV intake *decreased* compared to when FVs were pre-portioned exclusively. These authors conclude that, although salad bars appear to increase FV access, their installation might be insufficient to influence FV consumption for elementary students from lower income backgrounds [14]. Limitations of this study include the fact that it did not include comparison schools without salad bars, nor did it include schools from a broad range of socioeconomic backgrounds (as all schools were Title I).

A subsequent trial conducted by this same research team compared FV consumption in schools with salad bars to matched schools serving proportioned FVs only [15]. Results indicated that vegetable consumption was higher in salad bar schools; however varying patterns of FV selection and consumption were observed across school pairs, suggesting that school environment factors other than salad bar access influenced FV intake. In addition, schools with salad bars in this district also offered pre-portioned FVs on the serving line, precluding assessment of the independent influence of salad bars on FV consumption. Moreover, salad bar use varied widely (8–64%) between schools. This might be attributable to between-school differences in salad bar location, a factor demonstrated to impact usage [16]. In summary, there is an urgent need for longitudinal evaluations of school salad bars that include more schools (with greater sociodemographic diversity) and comparison groups, use robust scientific methods, assess dietary intake objectively, and place salad bars in a consistent location.

The current study extends prior research and addresses several of the methodological limitations noted above by randomly selecting schools (both Title I and non-Title I) receiving salad bars for the first time, matching them with schools serving pre-portioned FV only, and conducting a comprehensive, longitudinal evaluation that includes objective, validated assessments of FV intake within NSLP lunches. All FVs on the salad bars included in this trial *replaced* all fixed portion FVs on the serving line, and all schools operated under the NSLP policy requiring students to take at least one FV serving [5]. This study also extended previous work via its examination of FV both in combination (consistent with prior research) and separately, to determine if the effects of salad bars are consistent for fruits and vegetables. In addition, this trial assessed the relation between salad bar’s impact on FV consumption patterns and Title I school status, examined as a proxy for socioeconomic status, thereby providing important data about the potential effects of this intervention of those children most at-risk for inadequate FV consumption. It was hypothesized that schools with salad bars would have greater increases in both FV selection and consumption, and decreases in FV waste, compared with schools without salad bars. It was also hypothesized that salad bar students would select a greater variety of FVs.

## Methods

### Participants and setting

This investigation occurred in a large Mid-Atlantic school district, serving > 189,000 elementary school students (K-6th). This district installed salad bars into all 153 elementary schools over several years. The current evaluation was planned to be conducted over three academic years (2018–19 through 2021–22). Two years of data were collected prior to COVID-19 school closures in Spring 2020: post-assessments in 2 schools (year 2) and all of year 3 of data collection was not conducted as planned, yet as described below, this study remained well-powered to test study aims.

The school district determined the schedule of salad bar openings, which occurred throughout the school year. Schools receiving a salad bar during the study period, and for which both baseline and 4–6 week post data could be collected, were eligible for inclusion (e.g., schools with salad bars that opened after ~ March could not be included due to the summer holiday interfering with post-data collection). Given these criteria, there were 90 potentially eligible schools. Schools were first categorized based on Title I status (used here as a proxy for socioeconomic status) and race or ethnicity (percent of students from racial or ethnic minority backgrounds with higher obesity risk), yielding 4 categories: A = < 40% minority AND not Title I; B = 40–60% minority AND not Title I; C = 40–60% minority AND Title I; and D = > 60% minority AND Title I. The cutoffs applied were based on distributions observed in the district, in order to yield “lower”, “medium” and “higher” percentages of racial and ethnic minority students. One school within each category (A-D) that was scheduled to receive a salad bar (SB) that school year was randomly selected for inclusion. For each Salad Bar school, a Control school within the same category was randomly selected to serve as its matched pair. Potential Control schools were those that were scheduled to receive a salad bar the next school year. There were no “C” category schools available during the study timeline in Year 1, thus two C school pairs were selected in Year 2. Ultimately, seven school pairs were included (data from the 8th pair are excluded as no post data collection was conducted due to COVID-19). Although all schools were elementary level within the same district, there were some differences in grade levels served (e.g., K-3rd, K-5th, or K-6th). Thus, only trays from students in grades that were consistent across each pair were included. All students who participated in the NSLP on rating days were eligible. Complete study methods have been previously reported [17].

### Design

This district’s salad bar program was evaluated using a prospective, wait-list control design. The investigative team was not involved in salad bar installation—yet the district’s phased-in schedule permitted evaluation of a natural experiment. Each pair of schools was assessed on the same day at baseline, and again 4–6 weeks after the salad bar was installed (post), for menu consistency. Thus, there was one rating day per school at each time point. This duration of follow-up was selected due to its consistency with other studies [8], and to ensure that baseline and post assessments were conducted within the same school year (avoiding summer break and times the district does not allow research [start of the school year and during spring testing]), to enhance confidence that the same children were assessed at both time points. All schools in the district followed the same menu, with differences in offerings at post based on whether the school has a salad bar. In Control schools, FVs were served on the lunch line at both time points. In Salad Bar schools, FVs were served on the lunch line at baseline and on the salad bar at post. FV types offered differed between pairs at post, although the main entrées were consistent. Salad bar FV options included 4 vegetables (salad greens plus 3 other vegetables [e.g., cucumber slices, grape tomatoes, kidney beans]) and 3 fruits (e.g., halved bananas, orange slices, or apple slices). Schools without salad bars had ≥ 2 fruits and ≥ 2 vegetables on the menu each day. Additional and/or alternative FVs were offered based on availability. The study team documented which items were served at each school to determine any aberrations from planned menus across school pairs. All schools were operating under the NSLP guidelines, which require students to take a fruit and/or vegetable as one of their meal components at lunch.

Parent notification with the option to opt their child out of ratings and student assent were applied. All students in the school, regardless of participation, received a small incentive (e.g., FV-themed pencil or eraser) after post-assessments. Methods were approved by the Institutional Review Board of Virginia Commonwealth University. Training, and cafeteria and laboratory procedures are described in greater detail in Bean et al. [17], and briefly presented here.

### Plate waste procedures

Digital imagery plate waste procedures were applied [18]. On rating days, trained, masked assessors placed a label (with grade recorded) on assented students’ trays and took a digital image of the plated tray as students exited the lunch line (“pre-consumption” image). Labels were color-coded and numbered to track gender and facilitate subsequent rating by matching pre- and post-consumption images. Students left their trays on the table at the end of the meal. Assessors removed obstructions (e.g., napkins, utensils) and took a digital image of the post-consumption tray. All images were taken with iPads at ~ 45-degree angle.

Images were subsequently uploaded onto computers in the laboratory to prepare for rating. Trained laboratory raters (with excellent interrater reliability for starting portion [ICC = 0.94] and waste [ICC = 0.99]) simultaneously viewed the pre- and post-consumption images. Raters indicated: 1) which FV were selected, 2) starting portions for salad bar FV (to the nearest ¼ cup), and 3) the % plate waste for each item in 20% increments. Visual stimuli (pie charts) assisted raters in making plate waste judgments [19, 20]. At least 20% of trays were rated by two independent raters. Methods for estimating variable starting portions for FV from the salad bar have been previously validated and include using photographs and previously measured portions as a guide [14].

### Measures

#### Demographics

NSLP participation, % free and reduced-price meal participation, and school-wide demographics were obtained from the district and the Virginia Department of Education. Student school, gender, and grade were obtained from labels affixed to trays.

#### FV selection, consumption and waste

FV selection was identified from the images, and portions served, consumed, and waste were calculated. Pre-portioned FVs were served in standard ½ cup servings. Consistent with previously validated methods [18], rater estimates were used to determine the plated serving of salad bar FV to the nearest ¼ cup. These values were then converted to ½ cup portions (NSLP-defined portion) to facilitate comparisons across items. FVs were defined as whole FVs (excluding 100% fruit juice or FVs that were part of a combined food [e.g., tomatoes in sauce]). Consumption was determined by subtracting plate waste from the starting portion.

#### FV variety

FV variety was measured in two ways: 1) the number of different types of FVs selected and 2) the number of vegetable subgroups selected. Vegetables were categorized according to USDA/NSLP subgroups (dark green, red/orange, legumes, starchy, and other) [21], and the number of categories selected was calculated (ranging from 1–5).

### Statistical analyses

Analyses were conducted using SAS v9.4 [22]. Descriptive statistics were generated for all variables of interest. The data structure is hierarchical: students/trays are the unit of assessment and are clustered within schools. Thus, their treatment assignment is defined by the school’s assignment (Salad Bar or Control). To accommodate the nested structure of the data by allowing the specification of fixed and random effects and allowing correlated observations within schools, multilevel linear models were applied. Specifically, differences in selection, consumption, and waste of fruit, vegetables, and FV (combined), and differences in FV variety (number of types of FV and categories of vegetables) between group (Salad Bar and Control), time (baseline and post), and group-by-time interactions were evaluated. All models controlled for grade (as a proxy for body size) and school pair. To evaluate any differences by school-level socioeconomic factors, models were also stratified by Title I status. A Bonferroni correction was applied to protect Type I error rate, thus *p* < 0.002 was used to indicate significance of main effects and interactions. Effect sizes were also examined and interpreted as small (0.2), medium (0.5) and large (0.8) based on Cohen’s *d* [23].

Power and effect size of the multilevel models that were used to analyze the variety of outcomes in this investigation are a function of a number of parameters: 1) the number of clusters (J = 14 schools [7 Intervention and 7 Control]), 2) the cluster size (n = 230 lunches per school per time point), and 3) the ICC (estimated as 0.01, 0.05, and a much more conservative 0.10), which takes into account the correlated nature of these data and is the ratio of variability between clusters to the total variability [24]. Optimal Design Plus Empirical Evidence v3.01 software [25, 26] was used to calculate the minimum detectable effect size given the parameters above, α = 0.05, and a desired power of 80%. For ICCs of 0.01, 0.05, and 0.10, this study has 80% power to detect small to medium effect sizes of 0.19, 0.38, and 0.53, respectively.

## Results

See Table 1 for characteristics of included schools. Schools had a mean enrollment of 632 students (624 Salad Bar, 640 Control), with mean NSLP participation of 54% (55% Salad Bar, 54% Control), ranging from 31%-82% across schools. Overall, a mean of 42% students were eligible for free-and reduced-price lunch (42% Salad bar; 42% Control). Salad bar and control schools both offered a mean of 7 FV options at baseline. At post, control schools offered 7 FV and salad bar schools offered 8 FV options on average. Please see Table 2 for FVs offered at each school on rating days. The majority of entrées were matched across each pair. Aberrations from the planned menu included additional entrées or FVs served that were left over from the day prior or alternatives offered based on availability. Please see Table 3.

**Table 1:** Characteristics of elementary schools included in evaluation of school salad bars

	2018–2019 School Year						2019–2020 School Year							
	Pair 1		Pair 2		Pair 3		Pair 4		Pair 5		Pair 6		Pair 7	
	SB	C	SB	C	SB	C	SB	C	SB	C	SB	C	SB	C
Enrollment^a^	819	873	367	918	449	488	854	598	883	299	474	550	524	755
Grades^a^	K-6	K-6	K-3	K-3	K-5	K-5	K-6	K-6	K-3	K-3	K-5	K-5	K-6	K-6
% Minority^b^	< 40%	< 40%	40–60%	40–60%	> 60%	> 60%	< 40%	< 40%	40–60%	40–60%	> 60%	> 60%	40–60%	40–60%
Title I	No	No	No	No	Yes	Yes	No	No	No	No	Yes	Yes	Yes	Yes
NSLP rate (%)^c^	54.9%	34.4%	44.2%	41.3%	78.0%	82.0%	31.4%	42.3%	50.1%	53.5%	71.4%	69.9%	56.9%	52.2%
% FRPL eligible^d^	37.4%	7.6%	30.1%	33.0%	62.0%	65.5%	9.37%	31.8%	36.1%	45.2%	60.6%	75.0%	57.1%	33.8%
CEP^e^	No	No	No	No	No	Yes	No	No	No	No	Yes	Yes	No	No

*SB* Salad Bar, *C* Control

^a^Enrollment is based on grades included; only grades matched across pairs were included in analyses

^b^Includes racial and ethnic groups at higher obesity risk: Hispanic, Latino, Black/ African American, American Indian/Alaska Native, Native Hawaiian/Pacific Islander; used in create matching category based on school-wide demographics

^c^Average daily National School Lunch Program (NSLP) participation

^d^Percent of students eligible for Free or Reduced-Price Lunch (FRPL) based on family income

^e^School is participating in the Community Eligibility Provision (CEP), 100% of students receive free meals

**Table 2:** Fruits and vegetables offered at baseline and post for each pair of salad bar and control schools

	Baseline														Post													
Pair	1		2		3		4		5		6		7		1		2		3		4		5		6		7	
Condition	SB	C	SB	C	SB	C	SB	C	SB	C	SB	C	SB	C	SB	C	SB	C	SB	C	SB	C	SB	C	SB	C	SB	C
**Vegetables**																												
Baby carrots, fresh		x	x	x	x	x	x		x	x			x	x		x			x	x	x		x	x	x	x	x	x
Hashbrowns			x	x																								
Cucumber slices			x	x						x							x	x		x				x	x		x	
Broccoli florets, fresh	x	x		x					x	x			x			x	x	x	x		x		x				x	x
Green beans, canned			x		x				x	x	x									x				x				x
Corn	x							x			x	x			x							x						
Baked beans	x	x					x	x	x	x	x							x						x				
Side salad		x					x	x	x				x	x								x						x
Chili beans					x	x										x												
Tomato slices									x									x										
Salad mix															x		x		x		x		x		x		x	
Grape tomatoes															x		x		x								x	
Red pepper strips																	x				x		x		x			
Spiral potatoes								x						x								x				x		
Seasoned sweet potatoes							x		x	x														x				
Red beans																					x		x					
**Fruit**																												
Pineapple, canned			x	x						x			x					x				x		x		x		x
Orange, fresh (sliced, whole)	x		x	x	x	x		x	x			x	x	x	x		x	x	x	x	x		x	x	x	x	x	x
Banana, fresh (halved, whole, sliced)	x		x			x	x	x		x	x	x		x	x			x		x	x	x	x	x	x		x	x
Peaches, canned			x						x		x		x									x						x
Apple slices, fresh	x	x					x	x							x	x	x		x	x	x	x	x		x		x	
Pear slices, fresh																			x				x		x			
Pears, canned	x	x				x																						
Applesauce					x						x							x										
Mandarin oranges, canned							x	x				x				x						x		x				x
Apple, fresh (whole, sliced)						x		x	x	x								x								x		
Grapes																	x						x		x			
Cranberry sauce										x																		

At baseline, all schools served pre-portioned fruits and vegetables (FV). At post, salad bar (SB) schools served all FV on the salad bar and control (C) schools continued to serve pre-portioned FVs on the lunch line. Pairs were rated on the same day

**Table 3:** Entrées offered at baseline and post for each pair of salad bar and control schools

	Baseline														Post													
Pair	1		2		3		4		5		6		7		1		2		3		4		5		6		7	
Condition	SB	C	SB	C	SB	C	SB	C	SB	C	SB	C	SB	C	SB	C	SB	C	SB	C	SB	C	SB	C	SB	C	SB	C
**Entrée**																												
Peanut butter and jelly biteable^a^	x	x	x	x	x	x	x	x	x	x	x	x	x	x	x	x	x	x	x	x	x	x		x	x	x	x	x
Yogurt biteable^a^											x	x	x								x	x	x	x	x	x	x	
Chopped cheese salad^b^	x	x	x	x	x	x	x	x	x	x	x	x	x	x		x		x		x		x		x		x		x
French toast sticks (w/ or w/o turkey sausage)			x	x				x																				
French bread pizza	x	x																										
Teriyaki chicken with rice				x																								
Grilled cheese																												
Orange chicken with rice			x																x	x			x					
Hot dog on bun	x	x																										
Corn dog nuggets	x										x	x	x	x								x			x			
Pizza (cheese or pepperoni)															x	x												
Chicken tenders with rice							x	x									x	x	x				x	x	x	x		
Mini waffle bites & chicken tenders																	x	x										
BBQ chicken on pita																	x											
Broccoli cheese soup																		x	x	x								
Garlic knot																			x									
Beef teriyaki with rice			x																	x								
Tomato soup w/ garlic bread or breadstick											x	x								x							x	
Hamburger/cheeseburger on bun													x	x							x	x						
Cheese stuffed breadstick							x	x					x	x														
Eggo bites pancakes																						x						
Mini pancakes (w/ or w/o turkey sausage)											x	x		x								x		x				
Macaroni and cheese											x	x											x	x				
Chicken patty on a bun										x														x				x
Chicken on a biscuit									x	x															x	x		
Turkey ham and cheese on a bagel									x	x																		
Beef taco with pita									x	x								x										
Breakfast corn dog																							x					
Chicken on a bagel																										x		
Nachos					x	x																					x	x

Pairs of schools were rated on the same day

^a^Biteables were each packed with the main item (peanut butter and jelly sandwich or yogurt) and 2 additional sides that varied by school and time point (e.g., string cheese, cheez-its, soft pretzel, tortilla chips, shredded cheese, or goldfish)

^b^Entree salads were offered at all schools at baseline and at control schools at post; once salad bars were opened, they were no longer offered

Overall, *N* = 6,623 (*n* = 3,273 Salad Bar; *n* = 3,350 Control) trays were included (had a matched pre- and post-consumption image, able to be rated with no obstructions, grade recorded; see Supplementary Fig. 1 depicting the analysis sample and reasons for exclusions). Fewer than 1% of students opted out (either via parent opt out or in the lunch line). See Table 4 for characteristics of trays by student gender and grade for each school. About 2% of trays (*n* = 143) did not have a whole fruit or vegetable.

**Table 4:** N (%) for grade and gender^a^ by pair and school of student trays included in evaluation of school salad bars (*N* = 6,623: Salad Bar [*n* = 3,273] and Control [*n* = 3,350])

	2018–2019 School Year						2019–2020 School Year							
	Pair 1		Pair 2		Pair 3		Pair 4		Pair 5		Pair 6		Pair 7	
	SB	C	SB	C	SB	C	SB	C	SB	C	SB	C	SB	C
	Baseline						Baseline							
Grade														
K	19 (8.1)	44 (23.2)	22 (23.7)	80 (28.6)	46 (17.6)	63 (23.9)	28 (15.9)	26 (12.5)	89 (28.2)	35 (26.5)	51 (17.5)	53 (17.2)	20 (12.9)	5 (2.5)
1	28 (11.9)	28 (14.7)	32 (34.4)	73 (26.1)	56 (21.4)	53 (20.1)	27 (15.3)	18 (8.6)	82 (26.0)	22 (16.7)	48 (16.4)	49 (15.9)	19 (12.3)	22 (11.1)
2	41 (17.5)	26 (13.7)	20 (21.5)	66 (23.6)	47 (17.9)	49 (18.6)	32 (18.2)	34 (16.4)	81 (25.7)	39 (29.6)	65 (22.3)	58 (18.8)	19 (12.3)	14 (7.0)
3	35 (14.9)	37 (19.5)	19 (20.4)	61 (21.8)	40 (15.3)	24 (9.1)	14 (8.0)	44 (21.2)	63 (20.0)	36 (27.3)	48 (16.4)	51 (16.5)	30 (19.4)	45 (22.6)
4	48 (20.4)	15 (7.9)	--	--	39 (14.9)	37 (14.0)	28 (15.9)	35 (16.8)	--	--	37 (12.7)	47 (15.2)	29 (18.7)	37 (18.6)
5	43 (18.3)	29 (15.3)	--	--	34 (13.0)	38 (14.4)	21 (11.9)	21 (10.1)	--	--	43 (14.7)	51 (16.5)	20 (12.9)	39 (19.6)
6	21 (8.9)	11 (5.8)	--	--	--	--	26 (14.8)	30 (14.4)	--	--	--	--	18 (11.6)	37 (18.6)
Gender^a^														
Boy	117 (49.8)	94 (49.5)	51 (54.8)	128 (45.7)	127 (48.5)	134 (50.8)	102 (58.0)	106 (51.0)	156 (49.5)	70 (53.0)	150 (51.3)	163 (52.8)	84 (54.2)	96 (48.2)
Girl	118 (50.2)	96 (50.5)	42 (45.2)	152 (54.3)	135 (51.5)	130 (49.2)	74 (42.0)	102 (49.0)	159 (50.5)	62 (47.0)	142 (48.6)	146 (47.2	71 (45.8)	103 (51.8)
	Post							Post						
Grade														
K	56 (15.6)	41 (17.2)	32 (29.1)	82 (28.2)	42 (15.7)	62 (21.7)	25 (12.8)	36 (15.9)	82 (28.0)	33 (23.9)	53 (17.1)	66 (21.8)	24 (11.4)	24 (8.4)
1	47 (13.1)	36 (15.1)	35 (31.8)	79 (27.2)	51 (19.1)	54 (18.9)	32 (16.4)	22 (9.7)	83 (28.3)	28 (20.3)	61 (19.7)	61 (20.2)	25 (11.8)	26 (9.1)
2	50 (13.9)	32 (13.4)	28 (25.4)	71 (24.4)	47 (17.6)	59 (20.6)	34 (17.4)	32 (14.1)	72 (24.6)	40 (29.0))	62 (20.0)	54 (17.9)	32 (15.2)	14 (4.9)
3	38 (10.6)	46 (19.3)	15 (13.6)	59 (20.3)	42 (15.7)	30 (10.5)	16 (8.2)	42 (18.5)	56 (19.1)	37 (26.8)	52 (16.8)	38 (12.6)	42 (19.9)	59 (20.6)
4	64 (17.8)	20 (8.4)	--	--	43 (16.1)	43 (15.0)	28 (14.4)	33 (14.5)	--	--	38 (12.3)	49 (16.2)	36 (17.1)	50 (17.5)
5	55 (15.3)	42 (17.6)	--	--	42 (15.7)	38 (13.3)	22 (11.3)	29 (12.8)	--	--	44 (14.2)	34 (11.3)	25 (11.8)	54 (18.9)
6	49 (13.6)	21 (8.8)	--	--	--	--	38 (19.5)	33 (14.5)	--	--	--	--	27 (12.8)	59 (20.6)
Gender^a^														
Boy	182 (50.7)	102 (42.9)	55 (50.0)	140 (48.1)	123 (46.1)	148 (51.8)	84 (43.1)	118 (52.0)	140 (47.8)	71 (51.4)	155 (50.0)	152 (50.3)	109 (51.7)	134 (46.8)
Girl	177 (49.3)	136 (57.1)	55 (50.0)	151 (51.9)	144 (53.9)	138 (48.2)	111 (56.9)	109 (48.0)	153 (52.2)	67 (48.6)	155 (50.0)	150 (49.7)	102 (48.3)	152 (53.2)

*SB* Salad Bar, *C* Control

^a^Gender was determined by raters based on observations

**Fig. 1:**
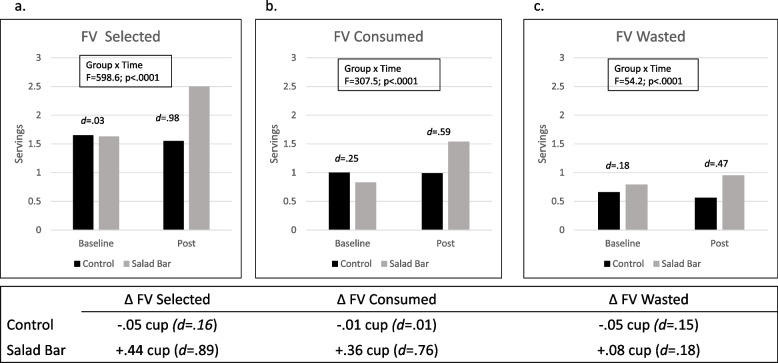
Fruit and Vegetable (FV) Selection, Intake, and Waste in Salad Bar and Control Schools. Note: Graphs display servings of fruits and vegetables (FV) selected (**a**), consumed (**b**) and wasted (**c**) in control and salad bar schools at baseline and post. Group by time interaction and between groups effect sizes (Cohen’s *d*) at each time point are shown. Tables below each graph display data as baseline to post change (Δ) in FV cups (1 serving = 1/2 cup) with the corresponding effect sizes (Cohen’s *d*) for the timepoint differences by group; *N* = 6,480

**Fig. 2:**
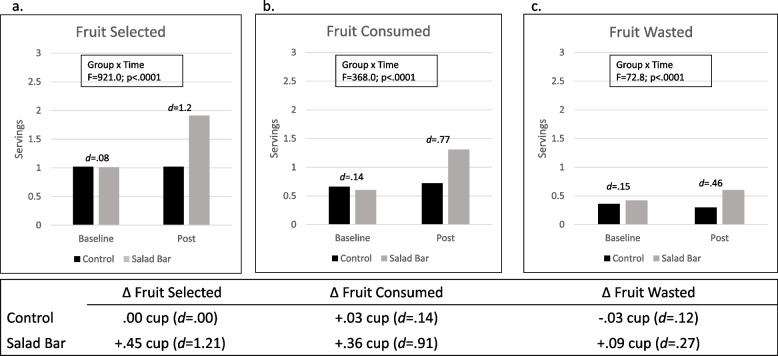
Fruit Selection, Intake, and Waste in Salad Bar and Control Schools. Note: Graphs display servings of fruits selected (**a**), consumed (**b**) and wasted (**c**) in control and salad bar schools at baseline and post. Group by time interaction and between groups effect sizes (Cohen’s *d*) at each time point are shown. Tables below each graph display data as baseline to post change (Δ) in cups of fruit (1 serving = 1/2 cup) with the corresponding effect sizes (Cohen’s *d*) for the timepoint differences by group; *N* = 5,670

**Fig. 3:**
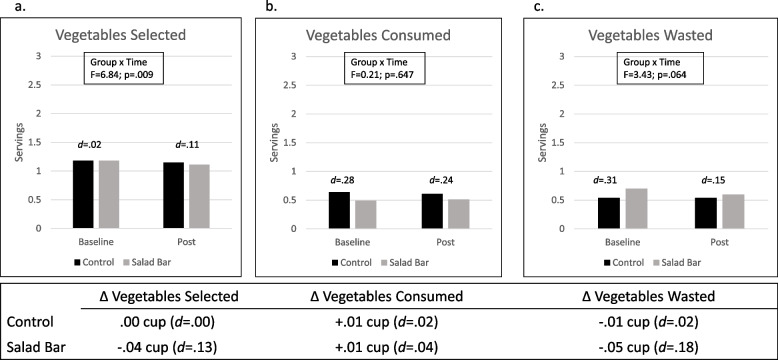
Vegetable Selection, Intake, and Waste in Salad Bar and Control Schools. Note: Graphs display servings of vegetables selected (**a**), consumed (**b**) and wasted (**c**) in control and salad bar schools at baseline and post. Group by time interaction and between groups effect sizes (Cohen’s d) at each time point are shown. Tables below each graph display data as baseline to post change (Δ) in cups of vegetables (1 serving = 1/2 cup) with the corresponding effect sizes (Cohen’s *d*) for the timepoint differences by group; *N* = 4,115

Figures 1, 2 and 3 display FV (Fig. 1), fruit (Fig. 2) and vegetable (Fig. 3) selection, intake, and waste, with corresponding between group (Salad Bar vs Control) effect sizes (Cohen’s *d*) at each timepoint (baseline and post). Graphs display each outcome in terms of servings, with the tables below each graph displaying the baseline to post change observed in cups of fruits and/or vegetables, to facilitate comparison to NSLP standards (1 serving = ½ cup). Effect sizes (Cohen’s *d*) for the change within each group are also presented. Please also see Supplementary Tables 1 and 2 for data from these models.

As shown in Fig. 1, Salad Bar students selected and consumed more FV at post compared with Control students (*p* < 0.0001). Specifically, salad bars increased FV selection by 0.44c and consumption by 0.36c, corresponding to large effect sizes (*d* = 0.76-0.89). At post, Salad Bar students consumed 1.5 servings (0.75c) of FV, whereas control students consumed ~ 1 FV serving. Salad Bar students had small increases in FV waste (+ 0.08c) and Control students had small decreases (-0.05c); yet both effect sizes were small (*d* = 0.15–18).

Figure 2 presents results for fruit and Fig. 3 presents results for vegetables, each examined separately. Results suggests that, when examined separately, effects were primarily driven by fruit (Fig. 2). Specifically, students in Salad Bar schools consumed 1.3 servings (0.65c) of fruit at post, compared with 0.7 servings in the Control schools (*d* = 0.77). The increase in fruit intake in Salad Bar schools (+ 0.36c) corresponds to a large effect size (*d* = 0.91), with no change in fruit intake observed in Controls. Small increases in fruit waste were observed in Salad Bar schools (+ 0.09c; *d* = 0.27), but not Controls. Salad bars did not change vegetable selection, intake, or waste—students in both Salad Bar and Control schools consumed ~ 1/4 c of vegetables at post. There was a significant group-by-time interaction for vegetables selected (*p* = 0.009), with decreased vegetable selection (-0.04c) in Salad Bar schools that was not observed in Controls, yet this effect was very small (*d* = 0.13) and is unlikely to be clinically meaningful (Fig. 3).

With respect to FV variety, Salad Bar students significantly increased the variety of FV selected, with no significant changes observed in Controls (Salad Bar: 1.81 ± 0.97 [baseline]➔2.09 ± 0.88 [post] FV types; Control: 1.81 ± 0.91 [baseline]➔1.77 ± 0.99 [post] FV types; F = 55.02; *p* < 0.001 for the group-by-time interaction). Neither Salad Bar nor Control schools significantly changed the number of vegetable subgroups selected (~ 1.3 vegetable categories at baseline and post for both groups).

When models were stratified by Title I status, similar patterns were observed between Title I and non-Title I schools, reflecting observations made in the overall sample. Please see Supplementary Tables 3 and 4 for data from these models.

## Discussion

Results indicated a link between salad bar installation and consuming 0.36 more cups of fruit, without corresponding changes in vegetable intake. Salad bar students also selected a greater variety of FVs, potentially leading to a greater range of nutrient intake. Although students in salad bar schools did not reduce their vegetable intake, they selected and ate more fruit than vegetables. The impact of salad bars on FV intake was similar across schools, regardless of their Title I status, suggesting that the installation of salad bars in school cafeterias could benefit children across the socioeconomic spectrum. This study extended prior work on the impact of salad bars on elementary students’ selection and consumption of FVs. Its design addressed several limitations of previous investigations in this area, including over-reliance on self-report measures of consumption and combined measurement of FV intake. It also included a large, socioeconomically, racially and ethnically diverse sample of schools, enhancing generalizability of study findings.

There was no significant change in vegetable waste with salad bars; both groups discarded about ¼ cup of vegetables at post. Salad bar schools wasted just over ¼ cup of fruit at post, representing a small (0.09 cups) but significant increase from baseline that was not observed in controls (fruit waste was ~ 0.15 cups at both time points in control schools). Although this increased fruit waste is small, school food waste reduction is an important priority, given the large scale of the NSLP, which operates in 99% of public schools in the United States. Indeed, a 2019 report found that 530,000 tons of food are discarded in schools each year (amounting to over $1.7 billion) [27]. Thus, although the increased fruit intake observed with salad bars is promising, additional strategies to help children with portion control and reduce fruit waste are needed to help address this concern.

Direct comparison to prior school salad bar investigations is somewhat limited given methodological limitations of previous reports (e.g., primarily self-report surveys [11–13], prone to response bias) [28]. Adams et al. [9] used objective plate waste methods similar to those applied here and did not find statistically significant increases in FV consumption in schools with salad bars—these findings conflict with the increased fruit intake we observed. However, that investigation was conducted prior to the current NSLP guidelines, thus might not be comparable given the current NSLP mandates that require a fruit or vegetable to be selected with each meal. The current study’s findings also differ from a longitudinal plate waste investigation in Title I elementary schools, which reported that FV intake decreased after salad bars were installed [8]—however, there were no comparison schools that did not have salad bars. Interestingly, a cross-sectional plate waste investigation within this same district found that vegetable consumption was higher in salad bar schools compared with control schools. Given the more rigorous design of the current investigation—longitudinal with control schools included—the increases in fruit intake that we observed, regardless of Title I status, might provide a more accurate understanding of the potential of school salad bars across sociodemographic position. Indeed, the current study methodology (longitudinal design, inclusion of a comparison group, objective plate waste methods) is a major strength, providing a clearer picture of the impact of salad bars on students’ FV consumption at that meal.

Given the known associations between FV consumption and chronic illness and mortality [29, 30], and data suggesting that children are not consuming the recommended amounts of these foods [31], current results are extremely promising. Previous research indicates that changes in fruit and vegetable consumption similar to those observed in the current study would yield significant public health effects. For example, a meta-analysis conducted with adults reported that each additional daily serving of fruits and vegetables is associated with reductions in all-cause mortality [29]. Moreover, results of a cost-effectiveness analysis of nutritional policies conducted in France indicated that an 80 g increase in daily FV intake (approximately 0.34cups) would have reduced deaths related to cardiovascular disease and cancer by 50% [32]. Thus, the value added by school salad bar installation could have important long-term effects on students’ health.

Future research should evaluate the impact of school salad bars (beyond 4–6 weeks) on consumption over a longer time period to assess the sustainability of the observed changes. In addition, it is vital to enhance vegetable consumption specifically, and future trials should investigate optimal intervention methods to achieve this goal. A recent review suggests interventions that made changes in the physical environment (e.g., placing vegetables at the beginning of the cafeteria line) were more successful in improving children’s vegetable consumption, compared with those that did not include environmental restructuring [33]. Although salad bars certainly represent an important alteration to the school physical environment, other aspects of successful interventions noted in this same review might enhance their impact and should be evaluated in future studies. These included providing feedback to children on their vegetable intake goals, working with parents and school staff to facilitate reinforcement of vegetable consumption, and having contact with students at least once per week for a period of six to twelve weeks regarding vegetable intake. Although these efforts would require additional resources, given the strong associations consistently identified between vegetable consumption and disease and mortality risk [29], they would have high public health significance. There is also a need to investigate lower intensity approaches, such as marketing promoting vegetable intake, a strategy with potential to influence dietary intake patterns [34, 35].

### Limitations

Despite its strengths, this study should be interpreted within the context of its limitations. First, this investigation was conducted within a single school district. Thus, its generalizability limited to that district and its model of salad bar implementation. Nonetheless, this district was very large and diverse in terms of race, ethnicity and socioeconomic status. Second, it is possible that the plate waste protocol applied could result in underreporting of very small quantities of FV selected. Salad bar FV were estimated to the nearest ¼ cup; thus, FV selection and consumption less than 1/8 cup would not be identified (multiplying any plate waste value × 0 starting portion = 0). To evaluate systematically the impact of this protocol on outcomes, we randomly selected ~ 10% of plates balanced across schools with salad bars (*n* = 207) and determined if there were FVs unrated that were closer to 0 than ¼ cup—items meeting these criteria were identified for 0.07% of trays (and were typically grape tomatoes and baby carrots). Thus, protocols were unlikely to yield significant changes to outcomes, yet should be considered when interpreting findings. In addition, FV selection and consumption could be impacted by which items were offered on rating days; although menus were matched (e.g., for the main entrée and other meal components), differences in offerings did occur. Future investigations could investigate optimal menu pairings and determine which FVs are more palatable for students. Although not included in this evaluation, obtaining student perceptions of salad bars, including what items are offered, can inform optimization of salad bars in schools.

## Conclusions

This study capitalized upon a natural experiment involving installation of school salad bars in a large, racially, ethnically, and socioeconomically diverse district. It used scientifically rigorous methodology, including random selection of schools, and objective plate waste measurement conducted by masked assessors. In addition, fruit and vegetable consumption and waste were assessed both in combination and separately, addressing limitations of prior work in this area. Its findings have important implications for the NSLP, particularly given that they suggest salad bars can benefit all children, bolstering the case for policy changes.

## Supplementary Information

Supplementary Material 1: Supplementary Figure 1. Order of exclusions from analyses to determine the final sample. Note: Double rated trays were retained in the sample until the final step to optimize the final analysis sample.

Supplementary Material 2.

## Data Availability

The datasets used in the current study are available from the corresponding author on reasonable request.

## Abbreviations

NSLP: National School Lunch Program
SB: Salad Bar
FV: Fruit and Vegetable
ICC: Intraclass Correlation
