# Supplementary material for: Impact of school salad bars on fruit and vegetable selection, intake, and waste in Mid-Atlantic elementary schools
Source: Int J Behav Nutr Phys Act. 2025 Feb 5;22:15. doi: 10.1186/s12966-025-01713-y (PMC11800604; doi:10.1186/s12966-025-01713-y)
Supplement: Supplementary file 1 — Supplementary Material 1: Supplementary Figure 1. Order of exclusions from analyses to determine the final sample. Note: Double rated trays were retained in the sample until the final step to optimize the final analysis sample. [file 12966_2025_1713_MOESM1_ESM.pptx]

## Slide 1
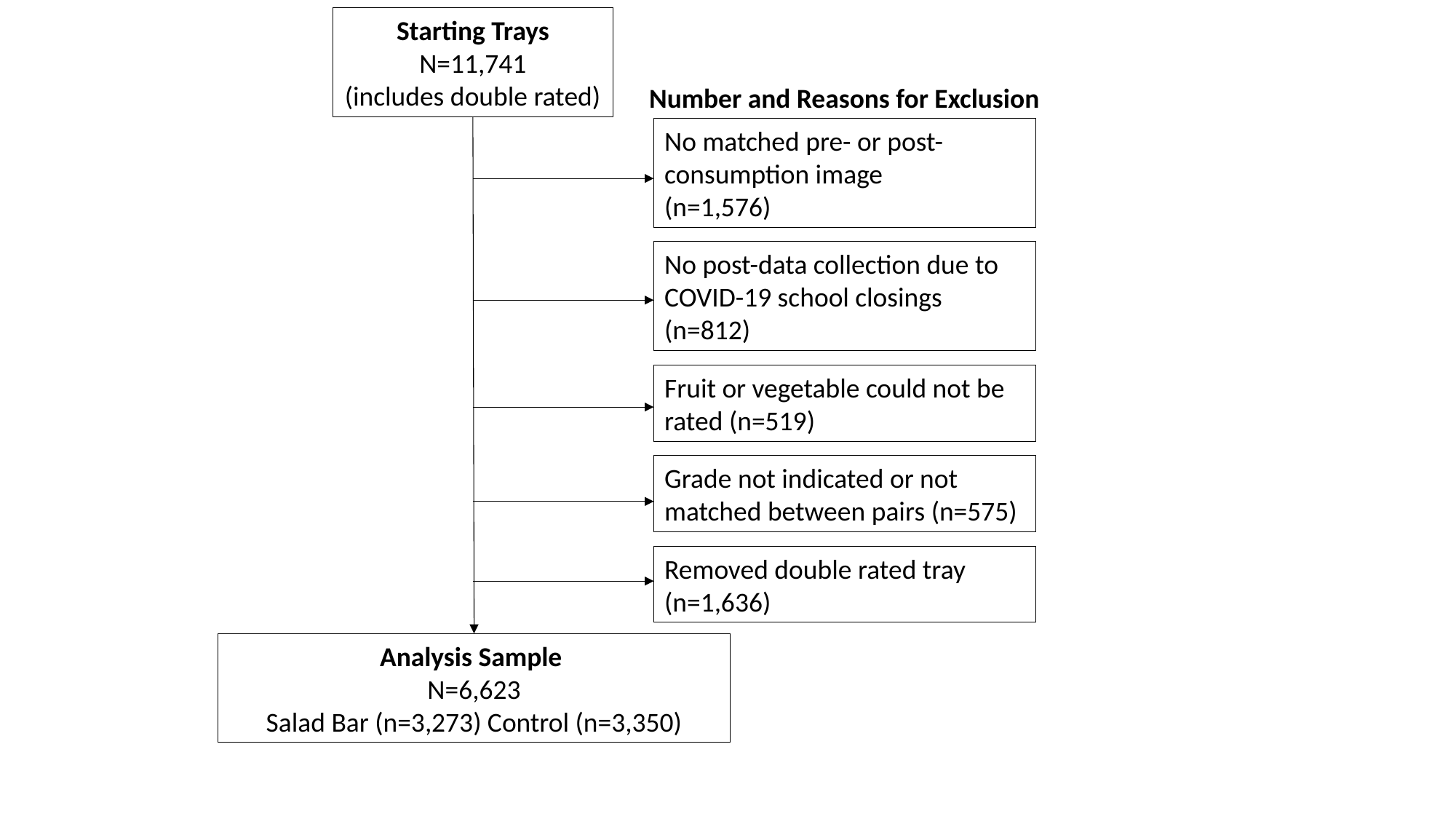

Starting Trays N=11,741
(includes double rated)
Number and Reasons for Exclusion
No matched pre- or post-consumption image
(n=1,576)
No post-data collection due to COVID-19 school closings (n=812)
Fruit or vegetable could not be rated (n=519)
Grade not indicated or not matched between pairs (n=575)
Removed double rated tray (n=1,636)
Analysis Sample
N=6,623
Salad Bar (n=3,273) Control (n=3,350)
