# Supplementary material for: Impact of school salad bars on fruit and vegetable selection, intake, and waste in Mid-Atlantic elementary schools
Source: Int J Behav Nutr Phys Act. 2025 Feb 5;22:15. doi: 10.1186/s12966-025-01713-y (PMC11800604; doi:10.1186/s12966-025-01713-y)
Supplement: Supplementary file 2 — Supplementary Material 2. [file 12966_2025_1713_MOESM2_ESM.docx]

Supplementary Table 1. Mean (SD) number of portions (1 portion = ½ cup) served, consumed, and wasted, for fruits, vegetables and for fruits and vegetables combined (FV), by group and time. Analyses include only those who were served fruit and vegetables, respectively (N=6,480).

| Treatment | Timepoint | Fruit  Served | Fruit Consumed | Fruit Wasted | Vegetables Served | Vegetables Consumed | Vegetables Wasted | FV  Served | FV  Consumed | FV  Wasted |
| --- | --- | --- | --- | --- | --- | --- | --- | --- | --- | --- |
| Control | Baseline | 1.02 (0.13) | 0.66 (0.42) | 0.35 (0.44) | 1.17 (0.40) | 0.64 (0.57) | 0.53 (0.52) | 1.65 (0.61) | 1.00 (0.68) | 0.66 (0.68) |
|  | Post | 1.02 (0.13) | 0.72 (0.41) | 0.30 (0.41) | 1.17 (0.39) | 0.65 (0.57) | 0.52 (0.51) | 1.55 (0.61) | 0.99 (0.66) | 0.56 (0.62) |
| Salad Bar | Baseline | 1.01 (0.11) | 0.60 (0.45) | 0.42 (0.45) | 1.18 (0.40) | 0.49 (0.51) | 0.70 (0.56) | 1.63 (0.64) | 0.83 (0.66) | 0.79 (0.74) |
|  | Post | 1.91 (1.05) | 1.31 (1.01) | 0.60 (0.82) | 1.11 (0.63) | 0.51 (0.61) | 0.60 (0.58) | 2.50 (1.23) | 1.54 (1.14) | 0.95 (1.01) |

Supplementary Table 2. Results (F [p-value]) of mixed models evaluating group and time differences and group*time interactions, accounting for pair and grade (N=6,480).

|  | Group | Time | Group*Time |
| --- | --- | --- | --- |
| Fruit Served | 784.96 (<.0001) | 898.62 (<.0001) | 921.06 (<.0001) |
| Fruit Consumed | 208.84 (<.0001) | 513.23 (<.0001) | 367.95 (<.0001) |
| Fruit Wasted | 134.18 (<.0001) | 18.32 (<.0001) | 72.83 (<.0001) |
| Vegetables Served | 1.84 (.176) | 6.05 (.014) | 6.84 (.009) |
| Vegetables Consumed | 44.49 (<.0001) | 0.03 (.857) | 0.21 (.647) |
| Vegetables Wasted | 33.18 (<.0001) | 4.01 (.046) | 3.43 (.064) |
| Fruits and Vegetables Served | 423.65 (<.0001) | 358.14 (<.0001) | 598.60 (<.0001) |
| Fruits and Vegetables Consumed | 76.81 (<.0001) | 292.64 (<.0001) | 307.48 (<.0001) |
| Fruits and Vegetables Wasted | 158.60 (<.0001) | 3.58 (.059) | 54.02 (<.0001) |

Supplementary Table 3. Mean (SD) number of portions (1 portion = ½ cup) served, consumed, and wasted, for fruits and vegetables (FV), by group and time, across Title 1 status. Analyses include only those who were served fruit and vegetables, respectively (N=6,480).

| Treatment | Timepoint | Fruit  Served | Fruit Consumed | Fruit Wasted | Vegetables Served | Vegetables Consumed | Vegetables Wasted | FV  Served | FV Consumed | FV  Wasted |
| --- | --- | --- | --- | --- | --- | --- | --- | --- | --- | --- |
| Not Title I | | | | | | | | | | |
| Control | Baseline | 1.03 (0.17) | 0.71 (0.42) | 0.32 (0.45) | 1.18 (0.39) | 0.60 (0.57) | 0.59 (0.53) | 1.82 (0.62) | 1.08 (0.74) | 0.73 (0.76) |
|  | Post | 1.02 (0.14) | 0.72 (0.42) | 0.30 (0.43) | 1.17 (0.40) | 0.62 (0.61) | 0.56 (0.49) | 1.64 (0.63) | 1.02 (0.79) | 0.62 (0.67) |
| Salad Bar | Baseline | 1.02 (0.13) | 0.62 (0.45) | 0.40 (0.45) | 1.26 (0.46) | 0.59 (0.55) | 0.67 (0.60) | 1.63 (0.68) | 0.89 (0.69) | 0.74 (0.73) |
|  | Post | 1.95 (1.08) | 1.34 (1.02) | 0.61 (0.87) | 1.14 (0.67) | 0.60 (0.66) | 0.54 (0.60) | 2.49 (1.25) | 1.60 (1.16) | 0.89 (1.02) |
| Title 1 | | | | | | | | | | |
| Control | Baseline | 1.00 (0.07) | 0.62 (0.41) | 0.39 (0.42) | 1.15 (0.40) | 0.70 (0.57) | 0.45 (0.48) | 1.47 (0.54) | 0.90 (0.60) | 0.58 (0.58) |
|  | Post | 1.01 (0.12) | 0.72 (0.39) | 0.29 (0.39) | 1.16 (0.37) | 0.69 (0.52) | 0.47 (0.52) | 1.45 (0.58) | 0.95 (0.61) | 0.50 (0.57) |
| Salad Bar | Baseline | 1.01 (0.09) | 0.57 (0.45) | 0.44 (0.45) | 1.10 (0.31) | 0.37 (0.45) | 0.73 (0.51) | 1.62 (0.60) | 0.77 (0.63) | 0.85 (0.74) |
|  | Post | 1.87 (1.02) | 1.27 (1.00) | 0.59 (0.77) | 1.08 (0.59) | 0.41 (0.54) | 0.67 (0.56) | 2.50 (1.20) | 1.47 (1.11) | 1.03 (0.99) |

|  | Group | Timepoint | Group*Timepoint |
| --- | --- | --- | --- |
| Not Title I | | | |
| Fruit Served | 370.23 (<.0001) | 481.21 (<.0001) | 511.31 (<.0001) |
| Fruit Consumed | 102.13 (<.0001) | 230.21 (<.0001) | 232.16 (<.0001) |
| Fruit Wasted | 62.87 (<.0001) | 25.34 (<.0001) | 31.71 (<.0001) |
| Vegetables Served | 0.46 (.499) | 9.27 (.003) | 7.15 (.008) |
| Vegetables Consumed | 1.86 (.173) | 0.20 (.655) | 0.54 (.461) |
| Vegetables Wasted | 0.70 (.403) | 5.37 (.021) | 2.78 (.096) |
| Fruit and Vegetables Served | 68.99 (<.0001) | 145.91 (<.0001) | 332.44 (<.0001) |
| Fruit and Vegetables Consumed | 32.86 (<.0001) | 114.57 (<.0001) | 170.32 (<.0001) |
| Fruit and Vegetables Wasted | 7.10 (.008) | 1.74 (.187) | 28.57 (<.0001) |
| Title I | | | |
| Fruit Served | 423.28 (<.0001) | 428.19 (<.0001) | 410.18 (<.0001) |
| Fruit Consumed | 105.58 (<.0001) | 293.91 (<.0001) | 134.60 (<.0001) |
| Fruit Wasted | 72.84 (<.0001) | 0.52 (.116) | 43.97 (<.0001) |
| Vegetables Served | 10.66 (.002) | 0.05 (.830) | 0.76 (.383) |
| Vegetables Consumed | 155.17 (<.0001) | 0.02 (.894) | 0.78 (.378) |
| Vegetables Wasted | 91.92 (<.0001) | 0.10 (0.749) | 2.66 (.103) |
| Fruit and Vegetables Served | 471.00 (<.0001) | 229.56 (<.0001) | 278.32 (<.0001) |
| Fruit and Vegetables Consumed | 47.39 (<.0001) | 186.07 (<.0001) | 139.88 (<.0001) |
| Fruit and Vegetables Wasted | 251.84 (<.0001) | 2.20 (.139) | 27.72 (<.0001) |

Supplementary Table 4. Results (F [p-value]) of mixed models evaluating group and time and group*time interactions, by Title I status. Pair and grade entered into all models as a covariate (N=6,480).
